# T-Cell Metabolism in Graft *Versus* Host Disease

**DOI:** 10.3389/fimmu.2021.760008

**Published:** 2021-10-29

**Authors:** Franziska Karl, Michael Hudecek, Friederike Berberich-Siebelt, Andreas Mackensen, Dimitrios Mougiakakos

**Affiliations:** ^1^ Department of Medicine 5, Hematology and Clinical Oncology, Friedrich Alexander University (FAU) Erlangen-Nürnberg, Erlangen, Germany; ^2^ Medizinische Klinik und Poliklinik II, Universitätsklinikum Würzburg, Würzburg, Germany; ^3^ Institute of Pathology, Julius-Maximilian University of Würzburg, Würzburg, Germany; ^4^ Deutsches Zentrum für Immuntherapie (DZI), Erlangen, Germany

**Keywords:** GvHD, T-cells, immunometabolism, GvT, allo-HSCT

## Abstract

Allogeneic-hematopoietic stem cell transplantation (allo-HSCT) represents the only curative treatment option for numerous hematological malignancies. Elimination of malignant cells depends on the T-cells’ Graft-*versus*-Tumor (GvT) effect. However, Graft-*versus*-Host-Disease (GvHD), often co-occurring with GvT, remains an obstacle for therapeutic efficacy. Hence, approaches, which selectively alleviate GvHD without compromising GvT activity, are needed. As already explored for autoimmune and inflammatory disorders, immuno-metabolic interventions pose a promising option to address this unmet challenge. Being embedded in a complex regulatory framework, immunological and metabolic pathways are closely intertwined, which is demonstrated by metabolic reprograming of T-cells upon activation or differentiation. In this review, current knowledge on the immuno-metabolic signature of GvHD-driving T-cells is summarized and approaches to metabolically interfere are outlined. Furthermore, we address the metabolic impact of standard medications for GvHD treatment and prophylaxis, which, in conjunction with the immuno-metabolic profile of alloreactive T-cells, could allow more targeted interventions in the future.

## Introduction

Allogeneic-hematopoietic stem cell transplantation (allo-HSCT) is a well-established and potential curative option for numerous high-risk hematological malignancies. Its therapeutic success, which depends on a mainly T-cell-driven Graft-*versus*-Tumor (GvT) reaction, is limited by the occurrence of Graft-*versus*-Host-disease (GvHD). Primarily (and like GvT) driven by alloreactive donor T-cells, with immune responses directed against foreign (host) antigens, GvHD can result in severe damage of host tissue, accounting for the majority of allo-HSCT-related morbidity and mortality (1, 2).

In GvHD treatment, T-cell responses are mitigated by immunosuppressive agents. However, increased susceptibility to infections, high mortality rates in steroid-refractory GvHD, and tumor relapses, emphasize the need for a deeper understanding of the T-cell pathobiology (1). As of to date, it is still challenging to balance GvT and GvHD. Consequently, novel and, more selective approaches that specifically target GvHD (but not GvT) while maintaining physiological immunity are required.

T-cell metabolism, function, and differentiation are tightly interconnected in health and disease (3). Interference with immune cell metabolism, a viable therapeutic approach in autoimmunity and inflammation, constitutes metabolic (T-cell) alterations as potential targets for disease control (4). In GvHD, chronic antigen stimulation results in robustly activated T-cells with a unique metabolic profile (5). A comprehensive characterization of such disease-specific metabolic (T-cell) signatures holds the potential for novel targeted therapeutic approaches counteracting GvHD.

**Figure 1:**
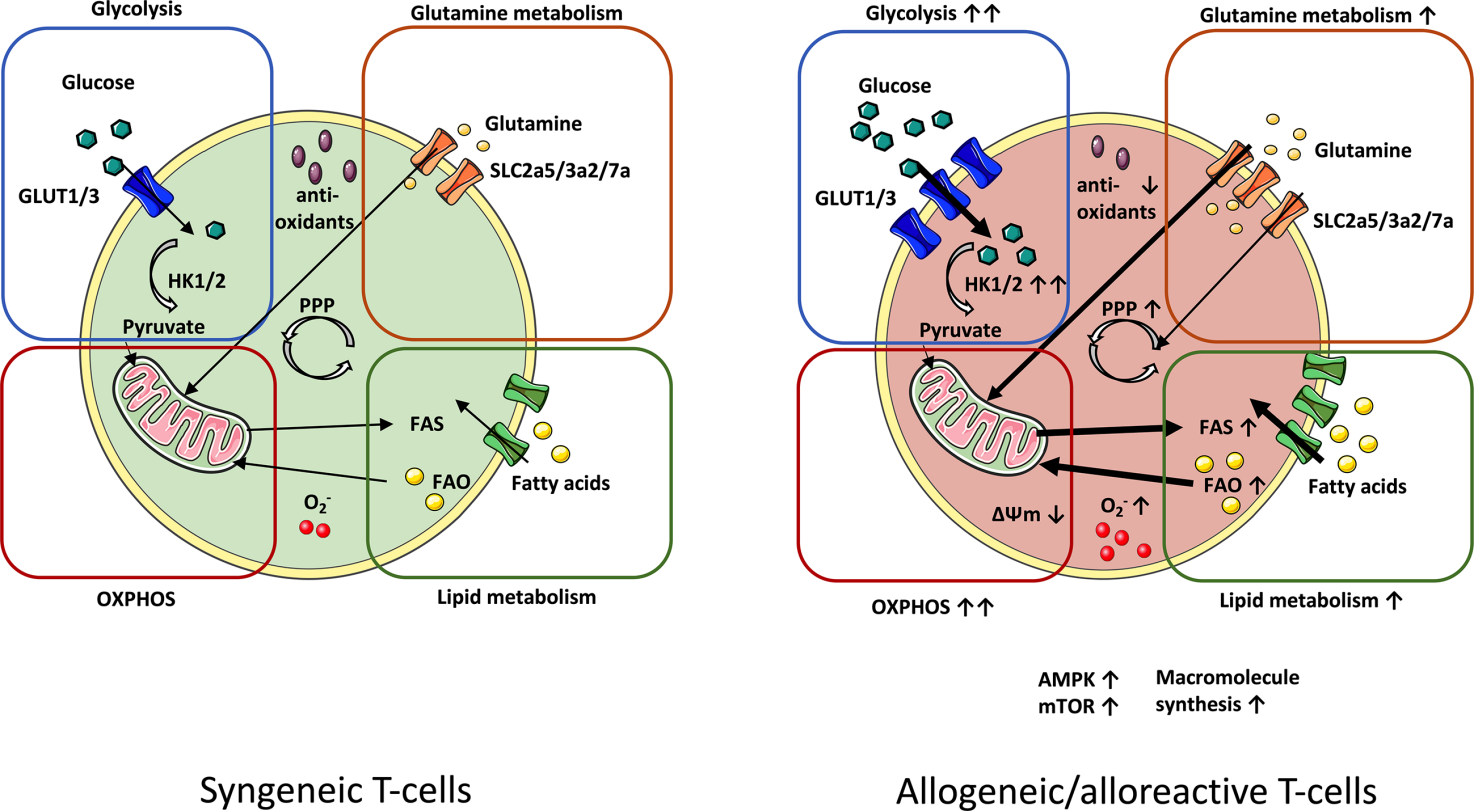
Metabolic profile of syngeneic *vs.* alloreactive T-cells. In order to meet their metabolic demands, GvHD-driving T-cells upregulate essential metabolic pathways. Glycolysis manifests as the principal source of energy in GvHD-causing T-cells. Fueling the TCA cycle with glycolysis-derived pyruvate reinforces increased OXPHOS activity in alloreactive T-cells. Enhanced OXPHOS potentiates production of ROS radicals (O_2_^-^), which is linked to lowered levels of antioxidants. Upregulation of glutamine metabolism further enhances OXPHOS by nourishing the TCA cycle with glutamine-derived a-ketoglutarate. Alloreactive/allogeneic T-cells display a superior lipid metabolism (FAS and FAO) and PPP-activity (fueled by glutamine as an anaplerotic source) as compared to syngeneic T-cells. Likewise, expression of the metabolic checkpoints AMPK and mTOR are elevated. Increased macromolecule synthesis complies with the demand of alloreactive T-cells for rapid cell growth and proliferation. AMPK, AMP-activated protein kinase; FAS, Fatty acid synthesis; FAO, Fatty acid oxidation; GvHD, Graft-*versus*-host-disease; mTOR, Mammalian target of rapamycin; OXPHOS, Oxidative phosphorylation; PPP, Pentose phosphate pathway; TCA, Tricarboxylic acid cycle.

Hence, this mini-review will provide an overview of known metabolic T-cell alterations in GvHD (summarized in **Figure 1**) and will illustrate potential intervention strategies (**Table 1**), which could ideally allow to selectively “turn-off” T-cell-mediated GvHD.

**Table 1:** Selected *in vivo* studies investigating the effect of immuno-metabolic interventions and conventional GvHD therapy on T-cell metabolism and on outcome in allo-HSCT.

Metabolic pathway		Type of intervention	Mechanism of action	ROA	Effect on GvHD	Species	Ref.
**Metabolic inhibitors (or affected pathways)**							
Glycolysis		2-DG	HK2 inhibition	systemic (i.p.)	none	mouse	(6)
		3-PO	PFKFB3 inhibition	systemic (i.p.)	reduction	mouse	(6)
		IL-1Ra antagonist	IL-1 receptor inhibition	*in vitro* treatment of donor T-cells	reduction	mouse	(7)
		GLUT1 KO in donor T-cells	GLUT1 inhibition	genetic	reduction	mouse	(8)
OXPHOS		BZ-423	F1F0-ATPase inhibition	systemic (i.p.)	reduction	mouse	(9)
		LYC-31138	F1F0-ATPase inhibition	systemic (oral)	reduction	mouse	(10)
		AMPK KO in donor T-cells	AMPK inhibition	genetic	reduction	mouse	(11)
		Metformin	AMPK activation	systemic (i.p.)	reduction	mouse	(12)
Lipid metabolism	FAS	ACC1 KO in donor T-cells	ACC1 inhibition	genetic	reduction	mouse	(13)
	FAO	Etomoxir	CPT1 inhibition	systemic (i.p.)	reduction	mouse	(14)
	FAO	Orlistat	LAL inhibition	systemic (i.p.)	reduction	mouse	(15)
	FAO	5-LO KO in donor leukocytes	5-LO inhibition	genetic	reduction	mouse	(16)
	FAO	Zileuton	5-LO	systemic (oral)	reduction	mouse	(16)
	SCFA signaling	GPR109a KO on donor T-cells	GPR109a inhibition	genetic	reduction	mouse	(17)
Glutamine metabolism		Glutamine administration	Substrate substitution	systemic (i.p.)	reduction	mouse	(18)
		Glutamine administration	Substrate substitution	systemic (oral)	reduction of GvHD related deaths	human	(19)
**Conventional GvHD therapy**							
N/A		GCR KO in donor T-cells	GCR inhibition	genetic	increase	mouse	(20)
Glycolysis		Rapamycin	mTORC1 inhibition	systemic (i.p.)	reduction	mouse	(6)
N/A		BEZ235	PI3K/mTOR inhibition	systemic (oral)	reduction	mouse	(21)
N/A		CC214-2	mTORC1/2 inhibition	systemic (oral)	reduction	mouse	(22)
Glycolysis		Echinomycin	HIF-1α inhibition	systemic (i.p.)	reduction	mouse	(23)
N/A		NFAT KO in donor T-cells	NFAT inhibition	genetic	reduction	mouse	(24, 25)

2-DG, 2-deoxy-D-glucose; 5-LO, 5-lipoxygenase; 3-PO, 3-(3-pyridinyl)-1-(4-pyridinyl)-2-propen-1-one; ACC1, acetyl-CoA-carboxylase-1; AMPK, AMP-activated protein kinase; CPT1, carnitine-palmitoyl-transferase; FAO, fatty acid oxidation; FAS, fatty acid synthesis; GCR, glucocorticoid receptor; GvHD, Graft-versus-Host-Disease; HIF1-α, hypoxia-inducible factor 1-alpha; HK2, Hexokinase 2; i.p., intraperitoneal; KO, knock out; LAL, lysosomal-acid-lipase; N/A, not available; NFAT, nuclear factor of activated T-cells; mTOR, mechanistic target of rapamycin; OS, overall survival; OXPHOS, oxidative phosphorylation; PFKFB3, 6-phosphofructo-2-kinase/fructose-2,6-biphosphatase-3; PI3K, phosphoinositide-3-kinase; Ref., reference; ROA, route of administration; SCFA, short-chain fatty acids.

## Dysregulated T-Cell Metabolism in GvHD 

In response to alterations of the tissue environment (e.g. nutrient fluctuations) and upon activation and differentiation, T-cells undergo metabolic reprogramming. This crosstalk between substantial metabolic- and immune-signaling pathways is regulated by metabolic checkpoints (e.g. Myc, HIF1-α, AMPK, mTOR) with immune-modulatory functions (26, 27). A detailed description of this regulatory framework is beyond the scope of this review and hence is covered elsewhere (28–33).

### Glucose Metabolism

Glycolysis is essential for T-cell activation, supporting T-cell growth and proliferation (5, 34). Importantly, besides that, glucose metabolism represents a key player in inflammation (35).

Several studies demonstrated increased glycolytic activity in T-cells when activated by allo-antigens (6, 36). In a murine bone marrow transplant (BMT) model, expression of the key glycolytic enzyme Hexokinase 1/2 and glucose transporter GLUT 1/3 were upregulated in the allogeneic in contrast to the syngeneic setup (6). A GLUT1-deficiency model further underscored the requirement of GLUT1 not only for T-cell proliferation and CD4^+^ effector T-cell (T_eff_) expansion but also for GvHD induction (8).

CD4^+^ T-cell-differentiated T_helper_ 1 (Th1), Th2, and Th17 T-cells are pathogenic in GvHD and preferentially utilize glycolysis (37). Glycolytic activity is crucial for Th1 and Th17-differentiation and blockade of Th17 induction is linked to decreased expression of glycolytic enzymes. Although CD4^+^ and CD8^+^ T-cells rely both on glycolysis, in the context of allo-HSCT there are subtle differences, with CD4^+^ T-cells being even more dependent on glycolysis than CD8^+^ T-cells. In fact, this increased (and potentially preferential) susceptibility of alloreactive CD4^+^ T-cells towards glycolytic interference could be therapeutically exploited (8, 37). Moreover, we observe this glycolytic shift of the CD4^+^ T-cell subset not only in models of acute but also of chronic GvHD, which has so far been very little investigated in terms of T-cell metabolism (38).

Targeting glycolysis has shown promising results in murine models: treatment with 2-deoxy-D-glucose (2-DG), a glucose-analog inhibiting initiation of glycolysis, diminished proliferation and activation of allogenic T-cells. However, that short-term *in vivo* treatment with 2-DG was not sufficient for GvHD-prevention and prolonged treatment resulted in severe toxicity. In contrast, 3-(3-pyridinyl)-1-(4-pyridinyl)-2-propen-1-one (3-PO), an inhibitor of 6-phosphofructo-2-kinase/fructose-2,6-biphosphatase-3 (PFKFB3), which represents a glycolytic rate-limiting factor, efficiently controlled GvHD (6). Differences in efficacy might be due to the underlying mechanism of action: while 2-DG specifically interferes with glycolysis initiation, PFKFB3-inhibition has more dispersed effects: in addition to promoting glycolysis, PFKFB3 is involved in cell cycle regulation, T-cell survival, and function (6, 39).

Nonetheless, interpretation of those findings should be approached cautiously. A recent study revealed metabolic reprogramming of donor T-cells by AML blasts. AML-derived lactic acid (LA) was found to be responsible for diminished metabolic activity (including glycolysis) in T-cells of relapsing allo-HSCT patients (40). Thus, one needs to take into consideration that targeting the metabolism of alloreactive T-cells might promote disease relapse. However, the metabolic dysregulation mediated by AML-derived LA might differ from the metabolic status resulting from specific glycolysis inhibition (discussed above), complicating the comparison.

Beyond this, the increased glucose uptake might have diagnostic implications. Non-invasive, *in vivo* monitoring of glycolytic activity by FDG-PET was shown to allow GvHD detection in murine models, with current efforts for clinical translation being ongoing (38, 41–43).

### Oxidative Phosphorylation (OXPHOS)

By fueling the tricarboxylic acid (TCA) cycle with metabolic products and generating ATP in the mitochondrial electron transport chain (ETC), OXPHOS is an efficient provider of energy (5, 29).

Increased OXPHOS and oxygen consumption (as compared to non-alloreactive/syngeneic T-cells) was detected in murine alloreactive T-cells. Enhanced superoxide production, decreased cellular antioxidant levels, and a hyperpolarized mitochondrial membrane potential suggest an abundant reactive oxygen species (ROS) production that is promoted by increased OXPHOS and an overall enhanced mitochondrial activity (9, 14, 44).

Targeting TCA- and ETC-activity with mitochondrial F1-F0-ATPase-inhibitors (BZ-423/LYC-31138), improved survival and limited GvHD in murine models (9, 10). As interference with the ETC by F1-F0-ATPase-inhibitors generates additional superoxide, cellular ROS burden further increases, leading to specific cell death of alloreactive T-cells. Thus, there is need to elucidate, whether the effects triggered by TCA/ETC blockade are ROS-dependent or the result of a rather bioenergetic deprivation (or both). Interestingly, PD-1-triggered ROS upregulation in T-cells is crucial for their subsequent metabolic modulation, as preceding PD-1-inhibition undermined the efficiency of F1-F0-ATPase-inhibition by LYC-31138. This effect might be explained by diminished ROS accumulation, which would (co-) facilitate apoptosis in alloreactive T-cells (45).

Noteworthy, the levels of TCA intermediates (including citrate, coenzyme-A) were found reduced in the allo- and syngeneic setup, which suggests pyruvate being predominantly converted to LA rather than TCA intermediates (6). However, OXPHOS-activity might change over time in reconstituting (T-)cells: in contrast to the general perception of increased OXPHOS activity in alloreactive T-cells at early timepoints after allo-HSCT (5-8 days), another study described similar OXPHOS levels in (murine) allogeneic and syngeneic T-cells at later timepoints (14 days post allo-HSCT) (6).

The key metabolic sensor AMP-activated protein kinase (AMPK) has been implicated as a driver of oxidative metabolism. Being reciprocally interconnected with the mechanistic target of rapamycin (mTOR), AMPK restricts anabolism (e.g. fat and protein synthesis) while improving catabolic pathways including OXPHOS and autophagy (5, 46). AMPK phosphorylation was found increased in murine alloreactive T-cells and genetic deletion of AMPK in donor T-cells showed protective effects against GvHD (11). In contrast, systemic administration of the AMPK-agonist metformin, promoted fatty acid oxidation (FAO) and alleviated GvHD (12, 47). Of note, metformin further inhibits complex I of the ETC, thereby interfering with T-cell metabolism also independent of AMPK activity (26).

Although the role of OXPHOS in alloreactive T-cells is still not conclusively clarified, ROS resulting from mitochondrial activity and required for proper T-cell activation as second messengers, may contribute to a continuous allo-activation upon BMT. In concurrence with this, targeting ROS pathways in preclinical models has reduced GvHD severity without impairing the GvT-effects (48).

### Lipid Metabolism

In addition to glucose metabolism, anabolism, and catabolism of fatty acids (FAs) regulate T-cell fate, proliferation, and differentiation of effector, memory, and Treg subsets (49).

Alloreactive T-cells exhibited increased FA-synthesis (FAS), with increased long-chain FA-transport and upregulated FAS-associated enzymes, alongside with enhanced FAO early after transplantation in a murine model (9, 14). Genetic interruption of *de novo* FAS *via* acetyl-CoA-carboxylase-1 (ACC1) inhibition in donor T-cells prevented acute GvHD and decreased glycolytic activity (13). This corroborates the notion that FAS is (amongst other functions) required for maintenance of glycolysis in allogeneic T-cells. Exemplary, the metabolic intermediate glycerol-3-phosphate (needed for FAS/triglyceride synthesis) also fuels glycolysis, which further underlines the complex interconnections between different metabolic pathways (50).

In addition, successful *in vitro* blockade of alloreactive T-cell expansion by sorafen A, a specific ACC1/2 inhibitor, might constitute a promising therapeutic strategy (13). Targeting FAO *via* etomoxir, which irreversibly inhibits carnitine-palmitoyl-transferase (CPT1), the enzyme responsible for shuttling FAs into the mitochondria, selectively affected alloreactive T-cells *in vitro* and *in vivo* (14). Moreover, etomoxir treatment inhibited PD-1-dependent increased respiration in murine alloreactive T-cells – a relevant consideration with respect to the emerging use of anti-PD1 therapies in the clinics, also in the context of allo-HSCT (45, 51).

Interfering with enzymatic FAO-regulation has additional implications in GvHD:

Lysosomal-acid-lipase (LAL) mediates intrinsic lipolysis by catalyzing the hydrolysis of cholesteryl esters and triglycerides in lysosomes and is required for physiological T-cell development and function (52). Pharmacological LAL-inhibition effectively controlled GvHD and preserved GvT-efficacy in a murine BMT model (15). Inhibiting 5-lipoxygenase (5-LO), an FAO-enzyme producing the proinflammatory leukotriene B_4_, by zileuton (clinically used for asthma treatment) or by transplantation of 5-LO-deficient leukocytes, improved survival and alleviated GvHD (16).

In addition to the dysregulated FA-metabolism, data from clinical trials suggests that dysbiosis of gut microbiota leads to abundance of FAs that are linked to GvHD outcomes. A multicenter-study with adults found high levels of circulating short-chain FAs (SCFAs; e.g. butyrate) to be associated with protection from chronic GvHD (53). Further, SCFAs might be used as a predictor of therapeutic efficacy against acute GvHD: patients responding to acute GvHD treatment displayed higher plasma SCFA concentrations compared to non-responders (54). This is underlined by a study based on infants, which found antibiotic treatment and reduced SCFA production to be linked to an increased gut GvHD risk (55). The finding, that genetic depletion of the butyrate/niacin receptor GPR109a in donor T-cells alleviates GvHD severity while preserving GvT activity, further underlines the interconnection of (alloreactive) T-cell metabolism, the microbiome, and GvHD, opening up new avenues for therapeutic interventions (17).

### Glutamine Metabolism

Glutamine is an anabolic energy source for DNA/RNA synthesis and an alternative carbon source fueling the TCA-cycle in activated T-cells [leading to production of the citrate precursor α-ketoglutarate (α-KG)] (56).

Alloreactive T-cells upregulate their glutamine-dependent TCA anaplerosis, which is reflected by increased glutamine, decreased glutamate, and increased levels of aspartate and ornithine (products of glutamate conversion to α-KG) (6, 10). Increased expression of enzymes controlling the conversion to glutamate (i.e. Gfpt1, PPAT, GLS2) as well as enhanced glutamine transport in allogeneic T-cells, emphasize the bioenergetic demand during expansion of reconstituting donor T-cells (6).

Conversely, emerging evidence suggests glutamine supplementation to be beneficial in view of GvHD. In a murine acute GvHD model, systemic glutamine administration boosted Treg frequency, limited pro-inflammatory immune responses, protected from GvHD, and improved survival (18). In patients, a glutamine-enriched nutrition after transplantation increased overall survival with a lower incidence of GvHD-related deaths (19). Hence, while glutaminolysis is increased in alloreactive T-cells, systemic glutamine administration seems to have a rather GvHD-protective effect. Therefore, the context- and cell type-dependent role of glutamine remains to be further deciphered.

### Pentose Phosphate Pathway (PPP)

As a part of the anabolic metabolism, the PPP is critical for nucleotide synthesis and is implicated in maintenance of the cellular redox balance (via NADPH regeneration) (30, 37).

Murine allogeneic T-cells exhibited an overall increased PPP-activity and enhanced levels of PPP-regulating enzymes [e.g. glucose-6-phosphate-dehydrogenase (g6dp), phosphogluconate-dehydrogenase (pgd)]. Although inhibition of g6pd by dehydroepiandrosterone (DHEA), did not affect donor T-cell expansion, it decreased the frequency of IFN-γ-secreting T-cells (6). The oxidative arm of the PPP is crucial for antioxidant formation, including the ROS-buffer glutathione (GSH). GSH is implicated in the inflammatory T-cell response and promotes T-cell expansion by promoting metabolic skewing of activated T-cells towards glycolysis and glutaminolysis, thereby meeting the metabolic requirements of proliferating T-cells (57). Chronic allo-stimulation leads to sustained nucleotide biosynthesis to support anabolic cell growth, resulting in decreased pyrimidine catabolism and exhaustion of the intracellular GSH pool (4, 6, 9). The aforementioned strengthened glucose-uptake in allogeneic T-cells (6), subsequently can fuel both glycolysis and PPP, turning the PPP into an integrating interface between glycolysis and macromolecule synthesis. In addition, it was shown that alloreactive T-cells utilize glutamine as an anaplerotic source to fuel the PPP (10).

## Conventional GvHD Therapy and Its Impact on T-Cell Metabolism

In addition to specific metabolic targeting, immunosuppressive drugs, commonly utilized for GvHD prophylaxis and treatment, can affect T-cell metabolism:

### Glucocorticoids (GCs)

Immunosuppression by GCs, the first line treatment against GvHD, has profound effects on T-cell development, differentiation, and function (58). GCs are regulators of glucose homeostasis and were shown to inhibit glucose uptake and glycolysis in T-cells (58–60), which is consistent with reduced GvHD-activity following restricted glucose uptake by donor T-cells (8).

Further, GCs were shown to suppress FAO-activity and FAO-related mitochondrial function *in vitro* and *in vivo*, which was accompanied by impaired memory T-cell formation and decreased tumor clearance *in vivo* (61). As memory T-cells are important drivers of GvT (without causing GvHD) (62), this interference with memory T-cell differentiation is of clinical interest (due to the potential increased relapse risk following allo-HSCT).

Interestingly, GCs seem to have differential metabolic consequences in the long-term: in a murine model, perinatal GC-treatment resulted in diminished CD8^+^ T-cell responses in adults, which was accompanied by increased OXPHOS-activity (63). Given the differentiation and reconstitution process of hematopoietic stem cells after allo-HSCT, in this scenario, those GC-triggered effects on T-cell immune-metabolism could be of significant relevance.

### Mammalian Target of Rapamycin (mTOR) Inhibitors

Inhibitors of the central metabolic checkpoint mTOR, are routinely used in transplantation medicine (26) (64). They have been introduced into the field of allo-HSCT and several clinical trials with the macrolide compound sirolimus revealed promising results for GvHD prophylaxis and treatment [reviewed in (65)]. Preclinical data evinced mTOR upregulation in alloreactive T-cells. In fact, mTOR can form two multiprotein complexes, mTORC1 and mTORC2 controlling its downstream effects including metabolic regulation, with mTORC1 being responsible for enhanced glycolysis in alloreactive T-cells and induction of GvHD (6). Blocking mTORC1 activity by sirolimus selectively attenuated glycolytic activity together with GvHD severity without affecting OXPHOS (6). Consequently, inhibiting phosphoinositide-3-kinases (PI3Ks) (upstream regulators of mTOR) simultaneously with mTOR, successfully prevented T-cell (allo-) activation and GvHD induction (21).

Novel dual mTORC1/2-inhibitors displayed stronger effects as compared to sirolimus in *in vitro* experiments leading to improved survival and reduced GvHD mortality *in vivo*. Importantly, T-cell responses against cytomegalovirus, an opportunistic virus that remains a major cause for morbidity in GvHD, were not affected (22).

The importance of glycolysis as an mTOR target in the GvHD context is further highlighted by the observation that direct blockade of hypoxia-inducible factor 1-alpha (HIF1α), an important regulator of aerobic glycolysis downstream of mTOR, with echinomycin, effectively reduced acute GvHD while preserving GvT by reducing glucose-dependent Th1 and Th17 cells and promoting Treg induction (23).

### Calcineurin Inhibitors (CNIs)

CNIs exert their immunomodulatory function by binding to immunophilins, resulting in calcineurin blockade. Upon activation, calcineurin, which is regulated by free cytosolic Ca^2+^, dephosphorylates its prime target nuclear factor of activated T-cells (NFAT), enabling NFAT translocation into the nucleus and subsequent NFAT target gene induction [crucial for T-cell activation and cytokine production (66)]. Since NFAT transactivates IRF4, HIF1α and GLUT3, CNIs additionally interfere with T-cell metabolism (26) (67).

Although CNIs, such as ciclosporin A (CsA) or tacrolimus (FK506), are extensively and successfully used in GvHD prophylaxis, dose-dependent negative effects on GvT-efficacy have been reported (68). Interestingly, selective NFAT targeting in T-cells reduced GvHD with maintained GvT-activity, when only one NFAT-family member was ablated (24, 25). This suggests that CNIs have broader (off-)target effects than sole and individual NFAT-inhibition. Additionally, a recent study proposed an overall NFAT-independent amelioration of GvHD by CNIs. By means of a genetic mouse model, this work showed, that dephosphorylation inhibition of the tyrosine kinase Lck by CNIs is primarily mediating their GvHD-suppressive effects (69).

However, despite that CNIs affect metabolic checkpoints, data on their metabolic impact in GvHD still remain limited.

CNIs suppress glucose metabolism-dependent activation of T-cells, thus retaining T-cells in a quiescent metabolic state (70, 71). Metabolic profiling of CsA-treated T-cells further revealed amino acid metabolism and PPP as targets (71). By the use of STIM1/2 double-deficient mice, Vaeth *et al.* demonstrated store-operated Ca^2+^-entry (SOCE) to regulate metabolic reprogramming *via* NFAT and the PI3K-AKT-kinase-mTOR pathway. Further, cell cycle entry of T-cells was found to be SOCE-dependent with SOCE-deficient T-cells being stuck in G0 phase, potentially explaining the regulation of T-cell proliferation by calcineurin blockade. These data propose a novel molecular mechanism by which SOCE, calcineurin, and NFAT control T-cell metabolism and function (70).

In clinical context, systemically administered CNIs additionally impact the glucose uptake in muscle and adipose tissue (crucial for glucose homeostasis), which may explain some of the CNI-mediated adverse systemic effects (26, 72). Moreover, novel cyclophilin-binding compounds bringing in new mechanisms of action might present an option for a more specific targeting with limited off-target effects (73).

### Inhibitors of *De Novo* Purine Synthesis

Similar to CNIs, data on the effect of the *de novo* purine synthesis inhibitors methotrexate (MTX) and mycophenolate-mofetil (MMF) on T-cell metabolism (in allo-HSCT) is rather limited. However, several *in vitro* studies implicate an effect of MMF on T-cell metabolism (i.e. *via* suppression of glycolysis), by interfering with AKT/mTOR signaling, thereby contributing to T-cell anergy and reduced T-cell proliferation (26, 72, 74). As of to date, no substantial data on potential MTX-elicited effects on T-cell metabolism are available [reviewed in (26, 72)].

Taken together, the vast evidence of studies on the immuno-metabolic impact of immunosuppressants address single agents. Combination therapies, as commonly utilized in clinical practice (2), that eventually lead to synergistic effects or even drive synthetic lethality (of alloreactive T-cells), remain largely unexplored. In one of the few studies, combination of sirolimus with CNIs has been shown to additively impact T-cell metabolism (71). Consequently, ideal (from the immuno-metabolic perspective) drug combinations for efficient therapeutic modulation have to be experimentally determined in the future.

## Concluding Remarks

In order to meet their metabolic demands, alloreactive T-cells upregulate essential metabolic pathways. With detailed knowledge on that metabolic signature of alloreactive T-cells in both acute and chronic GvHD, metabolic dissection of GvHD- and GvT-driving T-cells becomes more feasible. Via targeting alloreactive T-cells with customized metabolic inhibitors, this signature could be exploited therapeutically. Moreover, a bioenergetic profile, which specifically marks alloreactive T-cells, could be implemented as a novel GvHD-biomarker, consequently enabling intervention at early stages.

Emerging data on metabolic specificities of alloreactive T-cells, might also help to understand the underlying GvHD-pathobiology and complications frequently observed upon conventional GvHD treatments. In-depth bioenergetic characterization of the patients’ alloreactive T-cells could impact treatment decisions. Selecting drugs based on the fit of their mechanism of action and the T-cells’ actual metabolic profile, might lead to a more personalized approach, aiming at a secure and efficient treatment.

## Author Contributions

All authors listed, have made substantial, direct and conceptual contribution to the work, and approved it for publication.

## Funding

FK, MH, FB-S, AM, and DM were supported by a grant from the Deutsche Forschungsgemeinschaft (DFG, German Research Foundation), project number 324392634 - TRR 221.

## Conflict of Interest

The authors declare that the research was conducted in the absence of any commercial or financial relationships that could be construed as a potential conflict of interest.

## Publisher’s Note

All claims expressed in this article are solely those of the authors and do not necessarily represent those of their affiliated organizations, or those of the publisher, the editors and the reviewers. Any product that may be evaluated in this article, or claim that may be made by its manufacturer, is not guaranteed or endorsed by the publisher.
